# Coexistence of proboscis lateralis and multiple craniofacial, neurological, cardiac and spinal deformities: a one-of-a-kind case report

**DOI:** 10.1186/s12887-023-03882-w

**Published:** 2023-02-10

**Authors:** Asiya Kamber Zaidi, Aabiya Arif, Mehwish Butt, Sameer Saleem Tebha, Ishita Ray, Abubakr Yosufi, Puya Dehgani-Mobaraki

**Affiliations:** 1 Research Fellow, Associazione Naso Sano, Perugia, Italy; 2 MGM Medical College Indore (MP), Indore, India; 3 Department of Medicine, Ziauddin University, Karachi, Pakistan; 4 Department of Neurosurgery and Neurology, Jinnah Medical and Dental College, Karachi, Pakistan; 5 Department of Research, Kabul University of Medical Sciences, Kabul, Afghanistan; 6 President, Associazione Naso Sano, Perugia, Italy

**Keywords:** Proboscis lateralis, Bilateral colpocephaly, Cyanotic heart disease, Hemivertebra, Case report

## Abstract

**Background:**

Proboscis lateralis (PL) is an uncommon congenital facial deformity marked by the protrusion of a primitive tubular structure made up of skin and soft tissue that generally emerges from the eye's medial canthus and is associated with some craniofacial deformities. We report the first case of PL with multiple craniofacial, neurological, cardiac, and spinal anomalies.

**Case presentation:**

A full-term female baby delivered by cesarean section cried immediately at birth. The mother reported having a normal pregnancy but has a history of x-ray during her first trimester. The baby was born with a rare presentation of proboscis lateralis which was accompanied by multiple anomalies, including but not limited to bilateral colpocephaly, corpus callosum agenesis, complex cyanotic congenital heart disease, and hemivertebra of the T10 body.

**Conclusion:**

PL is an uncommon congenital condition that causes a variety of craniofacial abnormalities. Multiple additional defects affecting various organ systems should also be evaluated in a person diagnosed with PL.

## Introduction

Proboscis lateralis (PL), first described by Forster in 1861, is a rare congenital facial anomaly usually due to incomplete nose formation in utero. This abnormality is characterized by a primitive tubular structure composed of skin and soft tissue protruding from the facial surface. The medial canthus of the eye is the most commonly reported attachment site of PL; However, rarely has the PL been found attached to other regions such as the lateral canthus, lateral supraorbital ridge, mid-upper eyelid, midline of the root of the nose, and the chin [1, 2]. PL is usually a unilateral defect, but incidences of bilateral abnormalities have also been reported [2]. Even though PL can occur without any additional abnormalities, it is frequently associated with craniofacial congenital anomalies such as cleft lip and palate, encephalocele, holoprosencephaly, and abnormal development of the paranasal sinuses and orbit [3]. In this study, we present a rare case of a full-term baby born with PL, craniofacial abnormalities that are associated with PL, and multiple CNS, cardiac, and spinal congenital defects that have not been reported before along with PL.

## Case presentation

**Fig. 1:**
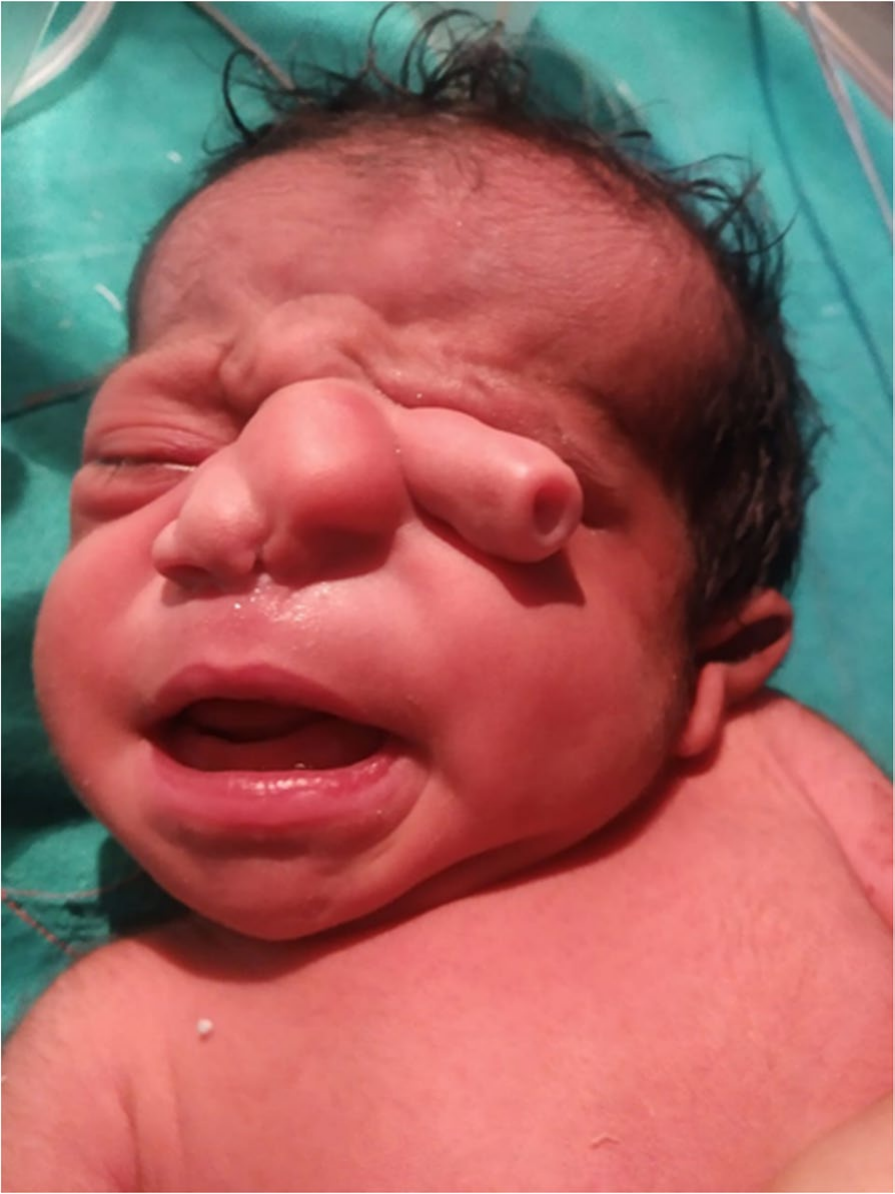
Clinical photograph of the patient showing a tubular trunk-like process arising from medial canthus of the left orbit

A five-day-old full-term female baby delivered by cesarean section cried immediately at birth. The mother had no comorbid conditions and described her pregnancy as normal. During the pregnancy, the mother had an X-ray of her leg, but she was unaware of her pregnancy at the time. There is no family history of congenital anomalies, and the baby is born to parents that are unrelated. The baby was born with a trunk-like appendage that was arising from the superomedial canthus of the left eye, 26 mm × 12 mm in size, with a small tract with Cerebrospinal fluid discharge. The left nasal cavity was hypoplastic with left anophthalmia. No cleft palate, cleft lip, or choanal atresia was noted. (fig. 1).

**Fig. 2:**
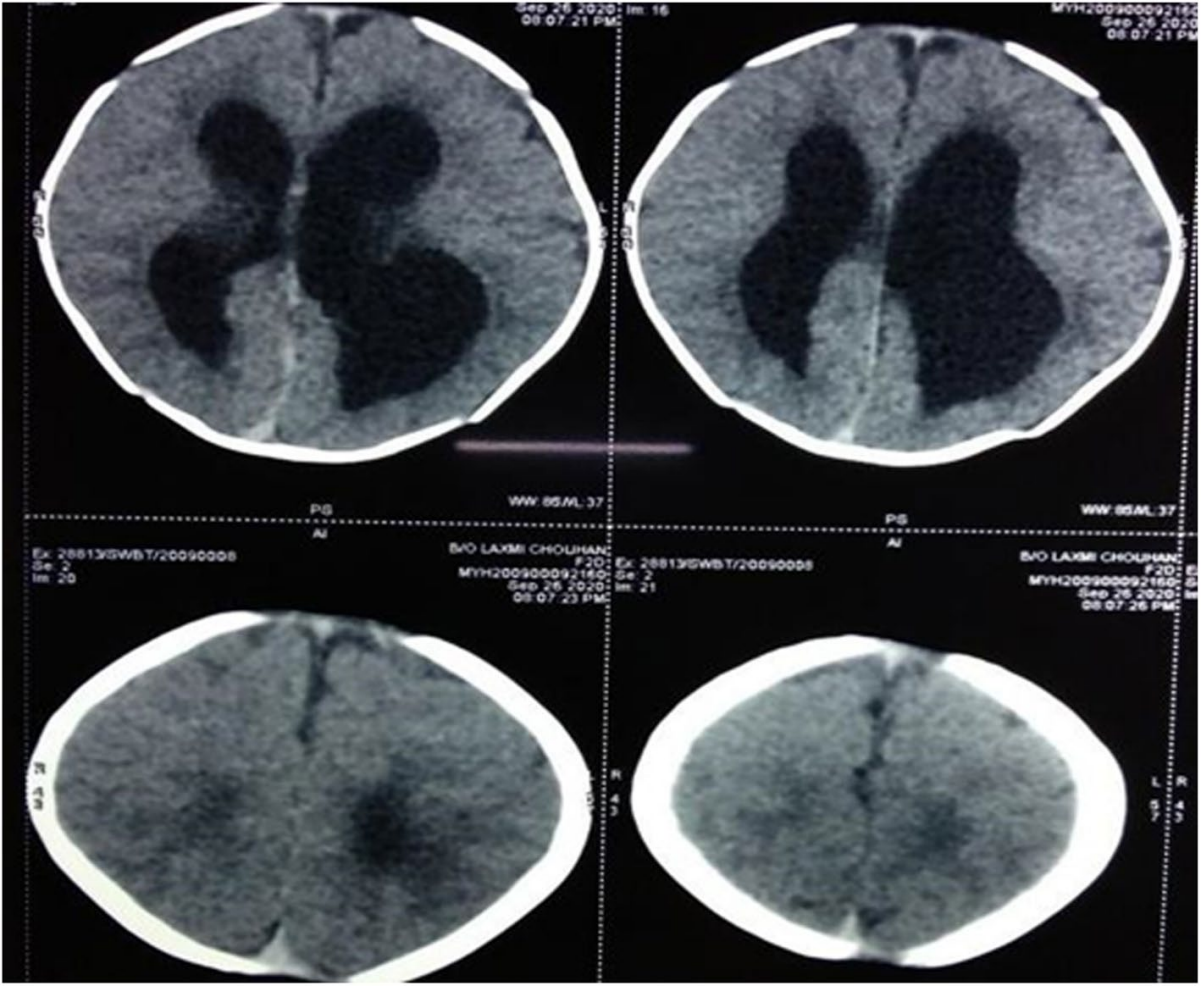
MRI brain

**Fig. 3:**
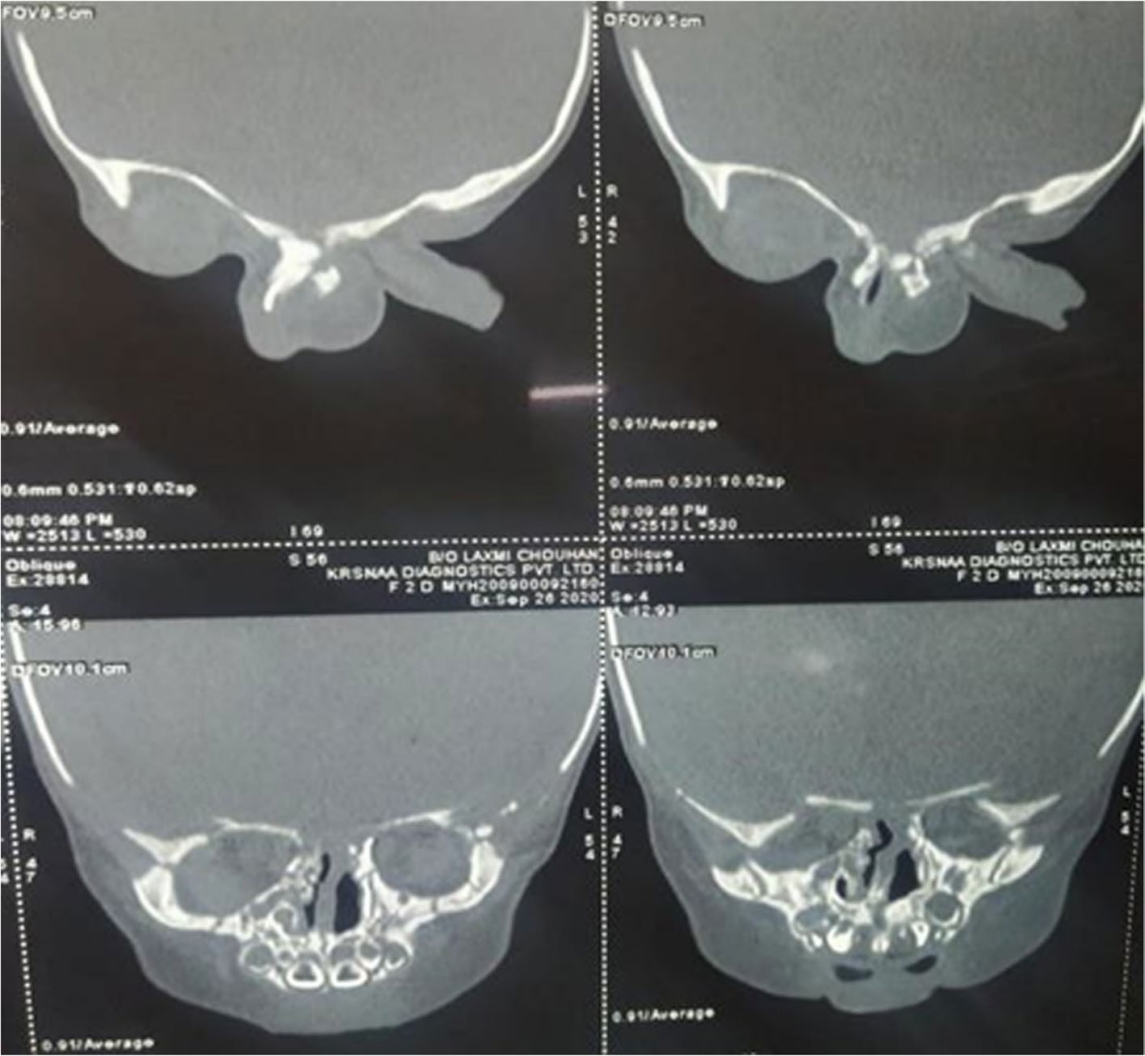
CT PNS

**Fig. 4:**
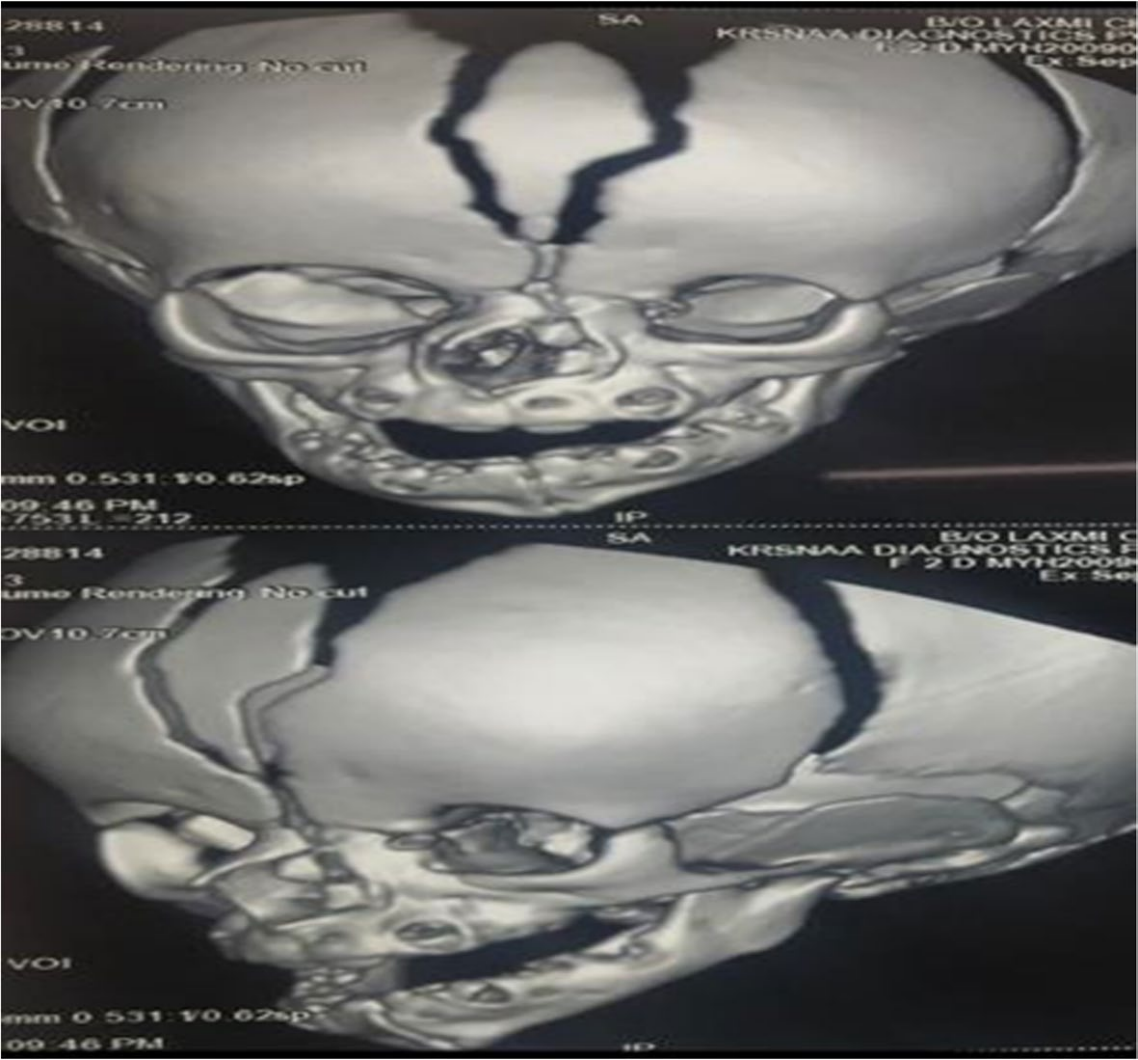
3D reconstructed image

A two-dimensional Echocardiography (2D echo) revealed complex cyanotic congenital heart disease, including double outlet Right Ventricle with a large ventricular septal defect of 8 mm, malposed great vessels, mild valvular right ventricular outflow tract obstruction (RVOTO) with a pressure gradient of 26 mmHg, small Patent ductus arteriosus (PDA) with a left to right shunt and confluent branched pulmonary arteries. Two ostium Secundum atrial septal defects (ASD) were noted of 4 mm and 25 mm with a normal biventricular structure and function. On Magnetic resonance imaging (MRI) (fig. 2) of the brain, mild hydrocephalus, bilateral colpocephaly (larger than normal occipital horns due to undeveloped white matter in the posterior cerebrum) with bilateral periventricular white matter hypodensities and corpus callosum agenesis were noted. Computed tomography (CT) imaging with a 3D reconstruction (figs. 3 and 4) of the face revealed minor bony defects of the left nasal bone, frontal process of the maxilla, and absent nasal turbinates.Bilateral maxillary and ethmoid sinuses were underdeveloped, to a greater degree on the child's left side. CT imaging of the thorax and abdomen revealed a hemivertebra involving the T10 vertebral body causing focal scoliosis with right-sided convexity.

Our patient’s parents deferred treatment and chose to wait till the child grows older.

## Discussion

PL is a rare congenital abnormality of the craniofacial anatomy caused by abnormal embryonic nasal development that occurs in 1 in 100,000 newborns [4]. The proboscis is a tubular structure that may have similar components to that of a normal nose, such as striated muscles, hair, sebaceous and sweat glands, etc. However, a proboscis does not have a cavity, rather, it has a narrow tube-like opening that usually does not contain the olfactory tract, unlike a normal nose [3].

There is not much known about the etiology behind the development of PL. The suggested pathophysiology involves the formation of defects in nasal placodes [5]. In the early fifth week of embryonic development, the nasal placodes covert into primitive nasal pits. It is thought that an error at this stage of development is what leads to the formation of PL [5]. PL is included in the list of congenital malformations named developmental field defects, which refers to major congenital abnormalities in a region that affects the development of surrounding areas, as PL is associated with a number of other congenital craniofacial defects [6]. The mechanism behind these related craniofacial defects is thought to be the indirect involvement of nasal placodes in the development of the face, and the palette since the development of the nose occurs simultaneously with these structures and the close association between primary brain vesicles and nasal placodes [7, 8].

Defects of the eyes, such as unilateral anophthalmia, coloboma of the iris, choroid, and lower or upper eyelid, have been reported in 44% of the cases, like in our case, the patient developed left anophthalmia. Facial bone anomalies, excluding the anomalies of the nasal bone, are found in 38% of the cases, and 19% of the cases reported structural anomalies of the central nervous system (CNS) [9].

In 1985, the Boo-Chai classification was developed, taking different possible features of proboscis and the other associated defects into account. The categorization split cases of PL into 4 groups. Group-1 is described as a person with proboscis but a normal nose, observed in 9% of the cases studied by Boo-Chai. Group-2 is proboscis, with nasal defects seen in 23% of the included population. Group-3 is taken as proboscis with a nasal defect along with defects of either the eye or ocular adnexa, seen in 47% of cases. Group-4 has all the features of group-3 with the addition of a cleft lip or palate, which was found in 21% of the cases in the research [4].

Later Sakamoto et al. added the component of intraorbital distance to the grouping of PL done by Boo-Chai, along with an addition of two more groups. Group 5 is defined as a PL with hypertelorism with encephalocele, a nasal, eye, and ocular adnexa defect, with either cleft lip or palate and Cerebro-oculo-nasal syndrome. Group 6 is similar to group 5 but presents with hypertelorism without encephalocele and, instead of Cerebro-oculo-nasal syndrome, presents with Holoprosencephaly [10].

Common CNS abnormalities seen with PL are encephalocele and Holoprosencephaly, but cases with other CNS abnormalities like meningoencephalocele, hydrocephalus, sphenoorbital basal cephalocele, corpus colosseum agenesis, arachnoid cyst, and brain stem asymmetry have been reported [5, 8, 11–16]. However, to our knowledge, there is no case, apart from our case, that presented with bilateral colpocephaly. Moreover, our case does not fit into any of the six groups created by Sakamoto et al., as our patient did not present with hypertelorism, encephalocele, or cleft lip or palate but developed multiple CNS anomalies along with PL.

The spinal deformity was reported only in one other case, where the patient had a malformation of the cervical vertebra along with PL [17]. Our case is the first to report a hemivertebra involving the T10 vertebral body and the second with a spinal deformity along with PL. In addition, this is also the first case to report multiple congenital cardiac anomalies along with PL.

The mother's exposure to radiation during the X-ray in the first trimester could be a contributing factor to the extensive list of abnormalities in the child. However, single diagnostic scans are considered safe by both the American College of Obstetricians and Gynecologists and the American College of Radiology. A single lower limb X-ray exposes the fetus to 0.0001 rad of radiation, whereas a minimum dose of 5 rad is estimated to be potent enough to cause congenital abnormalities [18, 19].

The management of PL requires A multidisciplinary approach involving several departments such as pediatrics, otorhinolaryngology, plastic surgery, anesthesiology, ophthalmology, radiology, and psychiatry services is essential for successfully managing functional and aesthetic aspects of PL. Cases involving the cardiovascular and nervous (Central and peripheral) systems, like the one we reported, required additional opinions from respective departments. The treatment of PL has long been a matter of debate. There is still no consensus about the time when treatment should start but early management in childhood is recommended to mitigate the psycho-social consequences of PL [2].

## Conclusion

PL is a rare congenital disorder associated with multiple anomalies. A person diagnosed with PL should be checked for multiple other anomalies involving other organ systems, even the ones not commonly associated with PL. As our patient had multiple anomalies like bilateral colpocephaly, hemivertebra of the T10 vertebral body, and multiple cardiac anomalies, which have previously never been reported in the literature. In addition, due to the effect of PL on the patient’s quality of life. Treatment should aim to achieve an acceptable appearance to ensure the patient’s psychological wellbeing and allow healthy social interactions. Parents need to be counseled to encourage early intervention in children born with PL.

## Data Availability

Not applicable.

## Abbreviations

PL: Proboscis lateralis
MRI: Magnetic resonance imaging
CT: Computed tomography
CNS: Central nervous system
